# Predicting Crashes Using Traffic Offences. A Meta-Analysis that Examines Potential Bias between Self-Report and Archival Data

**DOI:** 10.1371/journal.pone.0153390

**Published:** 2016-04-29

**Authors:** Peter Barraclough, Anders af Wåhlberg, James Freeman, Barry Watson, Angela Watson

**Affiliations:** 1 Centre for Accident Research and Road Safety – Queensland, School of Psychology and Counselling, Faculty of Health, Queensland University of Technology, Kelvin Grove, Queensland, 4059, Australia; 2 Empirica, Uppsala, Sweden; University of New South Wales, AUSTRALIA

## Abstract

**Background:**

Traffic offences have been considered an important predictor of crash involvement, and have often been used as a proxy safety variable for crashes. However the association between crashes and offences has never been meta-analysed and the population effect size never established. Research is yet to determine the extent to which this relationship may be spuriously inflated through systematic measurement error, with obvious implications for researchers endeavouring to accurately identify salient factors predictive of crashes.

**Methodology and Principal Findings:**

Studies yielding a correlation between crashes and traffic offences were collated and a meta-analysis of 144 effects drawn from 99 road safety studies conducted. Potential impact of factors such as age, time period, crash and offence rates, crash severity and data type, sourced from either self-report surveys or archival records, were considered and discussed. After weighting for sample size, an average correlation of *r* = .18 was observed over the mean time period of 3.2 years. Evidence emerged suggesting the strength of this correlation is decreasing over time. Stronger correlations between crashes and offences were generally found in studies involving younger drivers. Consistent with common method variance effects, a within country analysis found stronger effect sizes in self-reported data even controlling for crash mean.

**Significance:**

The effectiveness of traffic offences as a proxy for crashes may be limited. Inclusion of elements such as independently validated crash and offence histories or accurate measures of exposure to the road would facilitate a better understanding of the factors that influence crash involvement.

## Introduction

### The predictive power of traffic offences versus crashes

Researchers and various practitioners endeavor to identify the origins of crashes in order to save lives and reduce the subsequent significant emotional and financial burden. Unlawful driving behaviour and the resulting traffic offences are an important element of this work. Road authorities use them as a tool to reduce crash involvement, both as a general deterrent, and as a method to identify especially dangerous drivers. Similarly, researchers often use offences as a dependent variable in traffic safety studies [1]. This usage, however, rests upon the assumption that a fairly strong association between number of traffic offences and number of crashes actually exists. This is a common view amongst traffic safety practitioners. It will here be argued that this assumption is so far unverified, and that establishing what the true effect really is, is problematic.

First of all, it can be pointed out that although a reasonable number of studies have examined the association between crashes and traffic offences, and some researchers have reviewed this literature [2–4], no meta-analysis of this data appears to have been conducted. As will be described below, meta-analysis is necessary for the summary of large amounts of published data, both to calculate a mean effect over studies, but also to investigate whether effects systematically vary with other variables.

### Variation in effects for offences and crashes

A range of studies have examined the relationship between crashes and traffic offences, with some identifying an association between the two elements. Drivers involved in fatal crashes have been found to have more prior traffic violation convictions than non-culpable drivers [5,6]. Drivers understood to have previously incurred a high number of traffic offences have been strongly associated with being responsible for a subsequent crash [7,8]. Analysis of state-wide crash and traffic offence data from California, found earlier traffic convictions to be slightly more predictive of subsequent crashes than previous crashes [4,9–11]. A recent cohort study examining driver licence history of 10,063 victims of road trauma, found the number of prior traffic offences to be significantly associated with subsequent involvement in a severe crash, particularly alcohol-related road trauma [12]. Moreover, additional offences incurred following an initial crash was also associated with a greater likelihood of involvement in a subsequent crash. However a number of road safety researchers have questioned the effectiveness of traffic offences to act as a predictor of crashes [13–19]. In the present context, it can be pointed out that correlations between traffic offences and crashes can vary considerably (Pearson r of -.2[15] to .5[20]). The diversity of findings in this area, underscores the desirability of conducting a meta-analysis which, as described below, provides the ideal mechanism by which to interpret such differences.

However consideration should also be given to the extent that correlations between traffic offences and crashes may reflect a range of other factors instead of representing the actual relationship between driving style and a risk of crash involvement [18]. These factors could include: amount of time spent driving; effort directed towards detection of offences; exposure to the locations in which traffic violation are policed; drivers age and driving experience. In a longitudinal study tracking newly licensed drivers over nine years, Elliott et al [16] argued that the effectiveness of traffic offences to predict subsequent crashes would be reduced because driving behaviours changed as drivers became more experienced. Such an outcome would contribute to an expectation that studies using older drivers as subjects would produce smaller effects. Definitions of crashes and especially offences vary between studies, and particularly between countries. For example, in one jurisdiction a speeding ticket may be issued only when speed exceeds the limit by 5 km/h, but by 10 km/h in another. In this instance traffic offences represent different degrees of deviant driving behaviour. It is not possible to predict in which direction this phenomenon might affect effect sizes. Unfortunately the use of operationalisations as a moderator variable is not practical as it is infrequently provided in published papers and in many instances would not be possible to obtain, particularly for older publications.

Furthermore, methodological problems can influence the nature of results. In many instances small effects are not easily distinguishable from bias introduced by study design and analysis [21]. This in turn can influence attempts to ascertain an accurate population effect size. Common method variance in particular may affect results in this manner.

### Common method variance

Self-report data is widely used in the field of road safety research, including the measurement of crashes. This approach allows a larger number of crash types to be recorded, as archival data generally is restricted to more severe crashes. Self-reported crashes can include a wider range of crash types although crashes in which the driver has been killed cannot be recorded in this manner. Self-report surveys requesting driver history also tend to record all instances in which a driver has been involved in a crash, regardless of fault. Due to issues related to both memory recall and the relative rarity of crashes, a three year period is generally the most common timeframe to record this measure [22]. Self-reported data thus increases the variance of the crash variable, which should result in larger effect sizes. However, it is also known that there are limitations inherent with this approach, such as memory recall and method bias. Both of these could introduce systematic biases in the data, i.e. so-called common method variance (CMV). Research indicates that approximately 25% of all crashes are forgotten each year, with drivers more likely to report crashes that occurred closest to the time of the survey [23,24]. Method bias can reflect a tendency on the part of respondents to answer questions in a standardized manner, potentially distorting results.

Bias is also understood to be present in aspects of archival records relating to road safety. Injury severity is a factor that determines the likelihood of a crash being recorded by authorities, however police records of injuries sustained at crashes have been found to be inaccurate when compared with hospital records of the same incident [25]. Crashes involving cyclists have been found to be less likely to appear in police records than those involving motorised vehicles and there is also evidence to show that incidents involving pedestrians are underreported in police records [26–28]. Research also suggests that gender, ethnicity and remoteness of crash location contribute to under-reporting in police crash records [28–30]. Potential distortions of study findings due to common method variance have been a concern particularly in the social and behavioural sciences, and especially in studies involving self-reports such as questionnaires, surveys, and interviews [31]. Response bias introduced by CMV effects can distort findings by artificially strengthening or weakening the observed relationships between items of interest, resulting in unwarranted theorising and analysis of the relationships between the variables of interest [32,33]. The amount of variance attributable to method biases may vary by discipline and also in relation to the type of construct being examined[32]. For example method variance has generally been relatively low in the field of marketing (15.8%) and highest in the area of education (30.5%) [34].

### Examining CMV in road safety

Relatively little work has been undertaken within the road safety domain to assess the presence or otherwise of CMV effects. Generally these studies have involved the usage of ‘lie scales’ [35,36] or an equivalent tool to measure Social Desirability responding [37–41]. Previous research has found linkages between standard lie scales and self-reported crashes while at the same time observing that no such associations were found when measured against independently recorded traffic data [40,42–44].

More recently, the relationship between crashes and responses to the Manchester Driver Behaviour Questionnaire [45], after controlling for the number of crashes, was shown to be stronger when all the data was drawn from a single self-reported source than when survey responses were correlated against official driving records [46], thus indicating strong CMV effects. On the other hand, in a meta-analysis of personality as a predictor of crash involvement, measurements involving archive crash data actually produced larger effect sizes than that produced solely by self-report responses [47]. It should therefore not be assumed that all self-report scales are equally influenced by CMV effects.

Turning to offences and crashes, it could be expected that we would find a strong CMV effect for these data (i.e. self-reports would yield stronger effects when compared to records, after controlling for differences in restriction of variance). This prediction is due to the fact that these variables share many features, such as being relatively rare, negative events and liable to memory effects.

### Meta-analysis

Meta-analysis provides a method to efficiently summarise data from a large range of similar studies to calculate an estimate of an overall effect. Meta-analysis can identify trends and associations that may be too subtle to be otherwise detected or facilitate new interpretations of conflicting results [48,49]. In addition, a meta-analysis can reveal structural flaws and sources of bias in primary research and play a role in identifying promising research questions for future study [50].

One of the methods used in such analyses is to study the homogeneity/heterogeneity of the data, which is similar to the standard deviation of person-level data. In meta-data, however, it can be calculated whether the observed variance in effect sizes between studies is due to random error alone, or if other factors can be suspected to be at play (heterogeneity). If such is the case, an attempt is often made to explain the extra variance by the use of moderator analysis, which is similar to, for example, partial correlations. Moderator analysis simply detects whether effect sizes of different studies vary with other features of the studies, such as the year of publication. If moderators are associated with effect sizes, the calculation of a population effect size may become problematic. For example, the mean effect size in studies can be different for different age groups, indicating that a single population does not even exist, but several different ones. Thus, heterogeneity of data can profoundly affect how we interpret the available data.

Furthermore, heterogeneity of data may indicate that a few studies have results that are very deviant from the majority; outliers. This might indicate errors in these studies, and can be a reason for excluding their data points from the meta-analysis. Outlying values can be problematic, but not always detected. An outlying value can unduly affect the population estimate, and the size of the effect may be due to an error of transcription [1].

In studies on individual differences in crash record, there are always moderators present, especially restriction of variance in the crash variable [1]. This is due to the fact that different studies use different time periods for accident measurement, and populations with different risks. Therefore, there is a legitimate reason for effect sizes in such studies to vary in a manner that is beyond what can be expected from random error. This means that it is difficult to determine whether a certain value is really an outlier in a univariate analysis, as the standard cut-off of two standard deviations is not really applicable. A solution to this problem is to apply bi-variate outlier analysis [51], using the association between effect sizes and moderators, in the present case the means of the crash and offence variables (which are indicative of the degree of restriction of variance in these variables [1]), as well as the time periods of measurement for these variables.

In most meta-analyses, dissemination bias is considered as a potential problem, and therefore analysed in various ways. In the present paper, this was not the case, because no explicit theory about offences and crashes exists, and therefore there is no reason for writers and reviewers to favor certain results. Also, most results were obtained from papers which concerned other topics. Despite this, some analyses of possible bias were undertaken, as a standard precaution.

### Aims

The current study will apply meta-analytic techniques to explore and quantify the relationship between crashes and traffic offences within the general population and will examine and control for elements understood to potentially influence effect sizes, such as age, sample size, time period under investigation, regional factors and the type of data used. The basic hypothesis in relation to method bias is that effect sizes are inflated in studies in which self-reported data from a single source has been used. If CMV is inflating effects, self-reported data would be expected to yield stronger effects than those utilising archival records, after controlling for moderators [52–54].

## Methods

### Selection of studies

A literature search was undertaken using Google Scholar, ScienceDirect and Scopus for papers published prior to March 2015 which had reported or potentially captured the association between traffic offences and road crashes, with particular attention paid to the reference sections of relevant studies. A variety of search terms were employed including: crash; accident; traffic offence; violation; moving violation; tickets; fines; infringement; and citations. In addition to the studies which contained useful data, many papers were identified which appeared to have collected but not published information relevant for this undertaking. The authors or second authors of 116 papers were contacted by e-mail. Responses were received relating to 67 papers (57.8%), of which data relating to 37 studies was provided that could be included in the analysis, providing an overall successful response rate of 31.9%. Generally requests for data related to the provision of zero-order correlations between crashes and traffic offences, although other clarifications were requested relating to items such as the mean and standard deviation of the crash and offence variables, the study time frame and the mean age of the study participants. Occasionally effects were published, typically a Spearman’s rho or Cohen’s d, which required conversion to a Pearson’s correlation in order to be included in the analysis. This was done in line with appropriate formulas [55,56].

The relationship between the “crashes” and “offences” variables is naturally complex and could be presented in a number of ways. It is also acknowledged that generally these two variable types are discrete variables with non-normal distributions. However regardless of which metric or statistical method is employed, it would still reflect the limitations and biases of the original data. As almost all of the studies included in this study utilised correlations, Pearson’s *r* was selected as the principal metric used in the meta-analyses.

### Inclusion of papers

Generally a liberal approach was adopted in regards to the inclusion of papers in line with the approach favoured by Glass [57] although unique features of particular studies were noted. Studies involving all motorised vehicle types, including motorcycle riders, were included for consideration. A range of crash and traffic offence types were identified, with all considered for inclusion in the analysis, the obvious exception being traffic offence measures that included parking fines, as penalties of this type tend not to represent driving behaviour in any meaningful way.

On occasion studies provided effects relating to different crash types including; at-fault and not at-fault; active or passive and; minor or major crashes. Similarly in a small number of studies the traffic offence represented speeding fines or infringements issued from speed cameras exclusively. Whenever possible a figure that reflected a combination or total of these variables was used (for example Arthur & Graziano, 1996; Arthur & Doverspike, 2001; Scialfa, Ference, Boone, Tay & Hudson, 2010; Taylor & Sullman, 2009). If not, a two-step procedure was used, described below. Similarly, if results were broken down in terms of gender, a total figure was used if available. Occasionally crashes were recorded as a dichotomous variable. This tended to occur when the time period in question was rather short, such as six months or less. It was decided to include such studies as such a figure would not differ greatly to a figure representing a more exact total i.e. drivers are rarely involved in more than one crash in such a short period. Effort was also made to identify subsets from related studies to avoid duplication of data and to address the issue of correlations relating to a range of time periods. This was particularly the case in relation to data drawn from California driver record studies (see Gebers & Peck [58] for a summary of various time periods) but applied to other studies also. In these instances, providing that corresponding data relating to moderators was also present, effects drawn from the longer time periods were used.

In the current study three studies employed a predictive methodology: Daigneault, Joly & Frigon [17]; Diamantopoulou, Cameron, Dyte & Harrison [59]; and Stewart & Campbell, [60]. As the results from these studies were similar to more conventional studies (e.g., they did not appear as outliers) they were included in the analysis. Thus, papers were included in the data set if they reported data on the association between some type of traffic offences (excluding parking offences) and some measure of road traffic crash for drivers of all types of motorized vehicles. No restrictions were applied in terms of years of publication and no geographical or language limits were set, although in practice, almost all studies were written in English. At this stage, the dataset comprised values representing the different reported types of crash (for example major and minor crashes) or offence type. These figures were also used for the outlier analysis. However the meta-regression used only unique and study specific values, or independent values. This ensured that no sample was represented twice within this particular component of the analysis. Generally this resulted in the inclusion of one effect per paper unless data could be clearly differentiated as belonging to distinct groups within a study (for example men and women).

### Further selection of studies

While the outlier analysis is enhanced by the inclusion of a wide range of effects, a more conservative approach was adopted in relation to the selection of effects for inclusion in the meta-regression component of this study. If more than one effect per sample was available, preference was given to correlations from: the largest available sample size in repeated measures studies; a major crash over a minor crash as the former is a better match for crashes typically recorded by archival data; items for which a more complete set of information was available; at fault or “active” crashes in preference to those in which the driver was not deemed to be the cause of a crash (under the assumption that these should have the strongest association with offences [61,62]; and; the correlation which best reflected the focus of the original study. For example, work related crashes of professional drivers as opposed to those occurring during private vehicle usage.

In an effort to maximise homogeneity, studies with crash related offences were accepted for inclusion in the analysis. For example The California Driver Study of 1965 [14] drawing on archival data, reported a correlation between citations and crashes of 0.27. This figure dropped to 0.23 when offences related to a crash were excluded [14]. However, to promote greater study homogeneity, the former (higher) figure was included in the analysis.

### Moderating effects

In order to hold variance from these factors constant between studies, details relating to the mean number of' of crashes, the mean number of' of traffic offences, the timeframe for sampling crashes (these three variables all being measures of restriction of variance) and the mean age of study participants were collected. Data was also collected in relation to sample size, year of publication and the country in which the research was undertaken. This last component is of interest given the differences in laws and surveillance between countries, which again, may explain differences in findings. A brief description of the study sample was also recorded.

### Analyses

Several different meta-analytic methods were used to meet the aims of the study. This included calculations of mean effects under a random effects assumption (as it was presumed that the effects were not from a single population), moderator analysis and computations of homogeneity of data. All these analyses used the software Comprehensive Meta-Analysis v2 or v3. Additional analysis was conducted using SPSS. For other purposes, such as outlier analysis and exploratory moderator analysis, other methods were used, as described below.

Analyses were undertaken in different stages. In the first stage (section 3.1), all data was used, including dependent values. Thereafter (section 3.2), only independent values were used.

First, outlier detection analyses were run. A mean and standard deviation for the crash and for the traffic offence variables was calculated after these figures were annualised. Univariate outliers were identified if found to be more than two-standard deviations beyond these figures.

Detection of bi-variate outliers involved a calculation of the distance of each individual correlation from the regression line in a bi-variate association between crashes and offences (the Poom method). Once a measurement of the Euclidian distance for each point had been obtained, items found to be more than two-standard deviations beyond this figure were noted. This approach allows suspect values to be identified without necessarily being univariate outliers providing that a theoretically plausible association exists between the variables [46,51]. However, it is often not possible to be certain which value of the two yielded by an outlying point is causing the dissimilarity. Therefore, at least two bi-variate associations are required to identify a certain sample as yielding an outlying point if a certain value is to be considered for deletion. For example, if the point effect (offences/crashes) and crash mean was found to be a bi-variate outlier for sample A, and the crash mean and time period point was subsequently also an found to be an outlier, this would indicate some fault in the crash mean. Therefore, the crash mean in this sample would be deleted, but the other data retained. Thereafter, overall computations on all data were run. This included meta-analytic mean effects calculations, weighted by number of subjects, but also standard Pearson correlations between variables (the offences/crashes r, time period used in study, crash mean, offence mean, number of subjects, mean age in sample, year of publication, source of data and whether the result was published or not). Basically, these analyses identify possible moderator influences and other important effects which should be taken into account when interpreting the mean effects results.

Second, moderator analyses were applied to all data, using the variables tentatively identified as important in the previous step.

Third, where this was possible, mean effects analyses were run for each region separately, under the assumption that effects between countries are not really comparable, due to differences in laws and policing. Also, these analyses were run separately by source of data, thus creating a comparison of effects for self-reported and recorded data within each region.

Forth, on one occasion a “Sensitivity” analysis was conducted to allow for differences in time period. There is an expectation that a strong association exists between reported effects and the time period under consideration. To facilitate a more meaningful examination of the comparison groups, a sensitivity analysis was conducted if the statistical program was unable to satisfactorily control for time period under consideration. This process involved an additional analysis, in which studies with particularly long periods were excluded, results compared with the original analysis and differences noted. In this instance, studies with duration of over 6 years were temporarily put aside, allowing a sufficient number of studies to be included in the analysis while removing those with the potential to distort the findings. Results from both analyses are presented in the paper.

## Results

### Explorative analysis

#### Data

**Fig 1:**
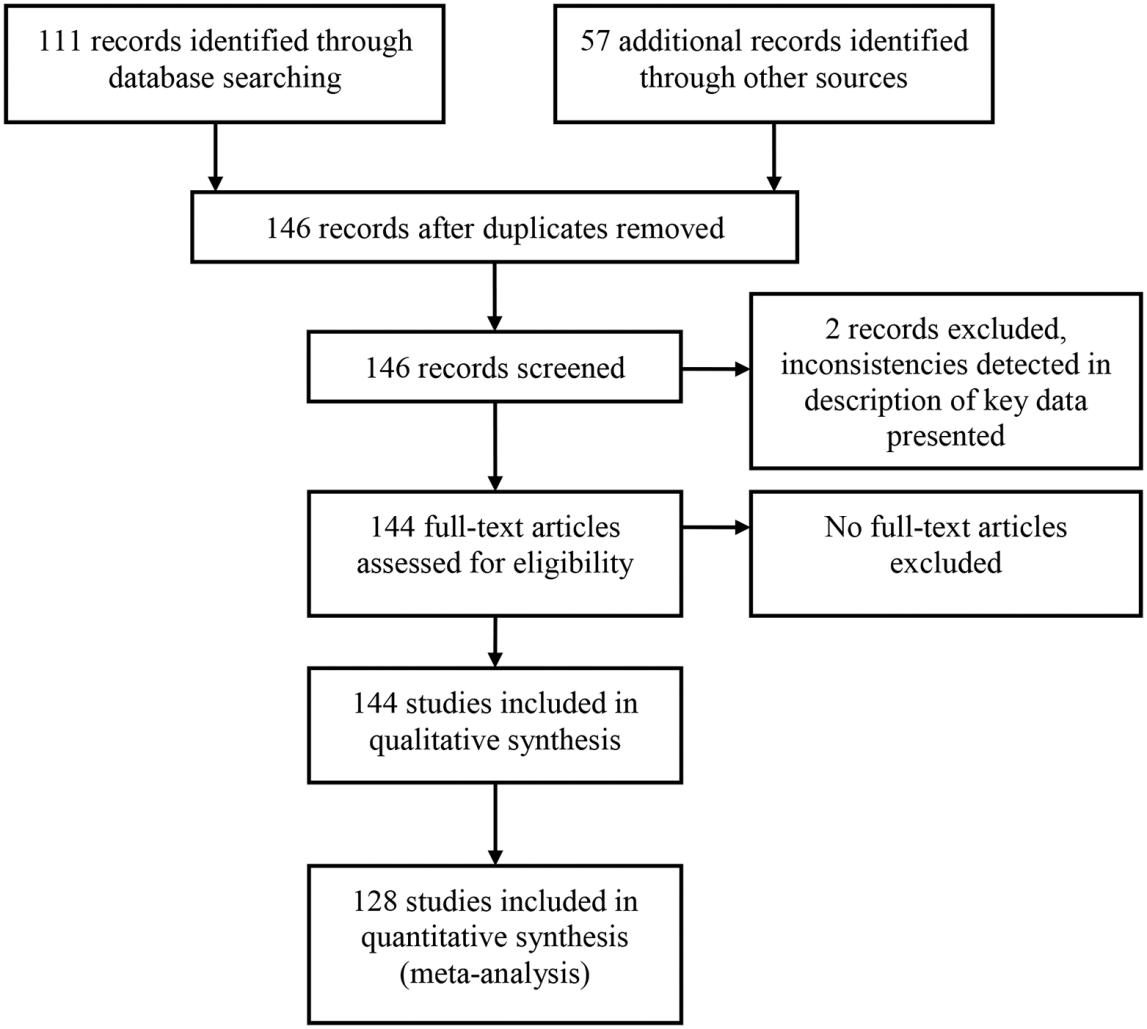
Study selection flow diagram.

After the removal of effects drawn from duplicated or overlapping samples, the search process identified a total of 144 effects drawn from 99 individual studies, with 74 studies providing self-report correlations and 27 studies providing data drawn from archival records. Data was retrieved from studies published between 1956 and 2015. Of the 144 correlations, 99 effects drew on self-report data with 45 effects produced using archival data. Two papers provided correlations for both data types (Barkley, Murphy, Du Paul & Bush [63]; Burns & Wilde [64]). A total of 57 effects were supplied by study authors upon request and four were obtained following conversion of the original effect or calculations utilising raw data provided within the published article. Authors also provided information or clarifications in relation to other variables related to the analysis. Fig 1 details the study selection process. A dataset consisting of all the effects noted above was used in outlier detection and preliminary analyses of associations between variables. In the analysis of mean effects and meta-regression, only independent study-specific values were used.

A breakdown of data by geographic area and type of data source are shown in Table 1. These figures represent the number of actual effects, as opposed to actual studies. For example Daigneault, Joly & Frigon [17] published four independent correlations in their 2002 study, with these accounting for half of the archival effects listed as being sourced from Canada. The category designated “Middle East” predominantly drew on studies conducted in Turkey but also includes one study of archival data from Israel. Dichotomous variables were used in three studies due to the short time period employed (3 months). Four self-report studies differentiated between major and minor crashes, recording data for both. A complete list of original data, country of origin, sample characteristics and other key variables from self-report studies is provided in Table 2 and for archival studies in Table 3[4,8,10,11,15,17,20,40,58–60,63–152].

**Table 1:** Distribution of retrieved correlations by region.

Region	Number of Studies	Self-report correlations	Archival correlations	Total correlations
US	24 Self-report 18 Archival 3 Combination	31	32	63
Canada	4 Self-report 4 Archival 1 Combination	4	8	12
Europe	22 Self-report	32	-	32
Australia & NZ	15 Self-report 4 Archival 2 Combination	20	4	24
Middle East	6 Self-report 1 Archival	8	1	9
Asia	1 Self-report	1	0	1
South America	2 Self-report	3	0	3
**Totals**	74 Self-report 27 Archival 6 Combination*	99	45	144

* Total number of individual studies is 100. Some studies supplied more than one data type.

**Table 2:** Data from self-reported road safety studies showing moderating variables and sample characteristics.

Study	Correlation	Crash Mean	Traffic offence mean	Time period (in years)	N	Age	Country	Sample
af Wåhlberg, 2010	0.15	0.58	0.73	3.2	7,497	21.7	UK	Young offending drivers
af Wåhlberg, 2010	0.10	0.72	0.34	12.7	1,225	31.9	UK	General drivers
af Wåhlberg, 2013*	0.07	0.32	0.736	3	4,807	42.6	UK	General drivers with recent traffic offence
af Wåhlberg, 2013*†	0.13	0.31	0.613	3	962	42.6	UK	General drivers with recent traffic offence
af Wåhlberg, 2013*	0.09	0.34	1.072	3	8,013	41.2	UK	General drivers with recent traffic offence
af Wåhlberg, 2013*†	0.00	0.34	0.806	3	407	41.2	UK	General drivers with recent traffic offence
Amado, Arikan, Kaça, Koyuncu & Turkan, 2014	0.19	0.83	3.1	5	158	37.5	Turkey	General drivers
Arthur & Graziano, 1996	0.36	0.92	2.18	4.3	477	20.3	US	University students and convenience sample
Arthur & Doverspike, 2001	0.48	1.13	1	3	48	23.9	US	University students
Arthur & Day, 2008	0.28	1.02	1.94	5	153	20.4	US	Young male university students and general drivers—Study 1
Arthur & Day, 2008	0.14	1.47	1.18	19.7	333	36.4	US	University students and general drivers—Study 2
Banks, 2008	0.15	0.24	0.09	1	679	42	Australia	Fleet vehicle drivers
Barkley, Murphy, Du Paul & Bush, 2002	0.68	1.9	11.7	4.5	88	21	US	Young drivers with ADHD
Barkley, Murphy, Du Paul & Bush, 2002	0.30	1.2	4.8	4.6	44	21	US	Young drivers control group
Cellar, Nelson & Yorke, 2000	0.30	0.5	-	10	202	-	US	University students—at fault crash
Cellar, Nelson & Yorke, 2000 †	0.19	0.5	-	10	202	-	US	University students—not at fault
Cestac, Paran & Delhomme, 2011*	0.15	0.41	0.17	3	3,002	22.3	France	Young Drivers
Conner & Lai, 2005	-0.10	0.17	1.79	0.5	1,188	38	UK	Participants in a driver improvement scheme—Wave 3
Conner & Lai, 2005 †	-0.05	0.09	1.8	0.5	1,047	39	UK	Participants in a driver improvement scheme—Wave 4
Constantinou, Panayiotou, Konstantinou, Loutsiou-Ladd & Kapardis, 2011	0.35	0.74	-	2.4	352	20.3	Cyprus	Young drivers
Crundall, Chapman, Poulter, Bibby & Clarke, 2012 *	0.04	0.56	1.22	2	225	46.8	UK	Truck drivers
Crundall, Chapman, Poulter, Bibby & Clarke, 2012 * †	0.08	0.05	1.22	2	225	46.8	UK	Private vehicle usage
Dahlen & Ragan, 2004	0.35	1.47	1.2	-	232	-	US	University students
Davey, Wishart, Freeman & Watson, 2007	0.22	0.12	0.24	1	443	44	Australia	Fleet vehicle drivers
Davey, Wishart, Freeman & Watson, 2007* †	0.03	0.09	0.12	1	439	44	Australia	Private vehicle usage
Deffenbacher, Oetting & Swaim, 2002†	0.05	0.12	-	0.25	290	19	Australia	Young drivers- minor crashes
Deffenbacher, Oetting & Swaim, 2002	-0.03	0.12	-	0.25	290	19	Australia	Young drivers -major crashes
Dejoy, 1992	0.24	0.6	0.91	3	136	20.6	US	University students—Spanish drivers
de Oña, de Oña, Eboli, Forciniti & Mazzulla, 2014 *	0.02	0.14	0.016	3	500	36	Spain	General drivers—Spanish sample
de Oña, de Oña, Eboli, Forciniti & Mazzulla, 2014 *	0.05	0.21	0.21	3	492	36	Italy	General drivers—Italian sample
Fischer, Barkley, Smallish & Fletcher, 2007*	0.29	1.88	6.12	3.6	85	21.1	US	Young drivers with ADHD
Fischer, Barkley, Smallish & Fletcher, 2007*	0.07	1.61	3.22	4.2	46	20.5	US	Young drivers—control
Fleiter, 2010 *	0.13	0.57	0.25	3	838	40.5	Australia	General drivers
Freeman, Wishart, Davey, Rowland & Williams, 2009	0.15	0.14	0.15	1	4,722	44	Australia	Fleet vehicle drivers
Freeman, Wishart, Davey, Rowland & Williams, 2009 *†	0.20	0.09	0.16	1	4,721	44	Australia	Private vehicle usage
Freeman, Barraclough, Davey, J, af Wåhlberg & Watson, 2013	0.26	0.39	1.9	3	249	37.4	Australia	General drivers
Furnham & Saipe, 1993	-0.20		-	-	73	28	UK	University students and convenience sample
Galovski & Blanchard, 2002	0.42	2.63	7.1	16.8	27	-	US	Aggressive drivers
Goldstein & Mosel, 1958	0.49	1.83	-	-	323	-	US	Convenience sample
González-Iglesias, Gómez-Fraguela, Romero & Sobral, 2012	0.32	1.41	1.22	5	292	40	Spain	General drivers—men
González-Iglesias, Gómez-Fraguela, Romero & Sobral, 2012	0.10	0.97	0.6	5	249	39	Spain	General drivers—women
Griffin, 2015*	-0.04	0.29	0.49	3	151	45.4	Australia	General drivers
Harris, Houston, Vazquez, Smither, Harms, Dahlke & Sachau, 2014*	0.26	0.51	0.72	3	1,181	20.9	US	University students
Hernández (2011)	0.14	0.37	0.59	2	487	30	Colombia	General drivers and university students
Jovanović, Lipovac, Stanojević & Stanojević, 2011*	0.15	0.41	0.53	3	260	32.5	Serbia	General drivers
Knee, Neighbors & Vietor, 2001	0.45	-	-	5	107	-	US	University students
Knouse, Bagwell, Barkley & Murphy, 2005*	0.33	0.26	0.77	1	88	31.9	US	Drivers with ADHD and control group
Lucidi, Giannini, Sgalla, Mallia,Devoto & Reichmann, 2010*	0.29	0.14	0.103	0.66	1,008	18.3	Italy	Young drivers
Lucidi, Mallia, Lazuras & Violani, 2014*	0.02	0.08	0.273	1	485	68.1	Italy	Older drivers
Lourens, Vissers & Jessurun (1999)*	0.20‡	0.07	0.6	1	1,190	-	Netherlands	Older drivers
McGuire, 1956a	0.03	1.72	-	-	67	-	US	Drivers with recent crash
McGuire, 1956a	0.30	1.93	-	-	57	-	US	Drivers with recent crash
McGuire, 1972	0.24	-	-	2	1,481	-	US	Recently licensed drivers
McGuire, 1972	0.32	-	-	2	1,480	-	US	Recently licensed drivers
Mesken, Hagenzieker, Rothengatter & de Waard, 2007*	0.00	0.7	1.8	3	44	45.9	Netherlands	General drivers
Møller & Haustein, 2014*	0.13	0.13	0.07	0.5	1,041	23	Denmark	Male drivers aged 18 and 28
Ouimet, Morton, Noelcke, Williams, Leaf, Preusser & Hartos (2008)*	0.22	0.88	0.71	1	2,334	19	US	Teenagers
Ouimet, Morton, Noelcke, Williams, Leaf, Preusser & Hartos (2008) *	0.11	0.25	0.29	5	2,280	47	US	General drivers
Özkan, & Lajunen, 2005	0.38	2.08	2.37	7.6	306	29	Turkey	General drivers
Özkan, Lajunen, Doğruyol, Yıldırım & Çoymak, 2012* active crash	-0.02	0.8	0.63	3	451	33.9	Turkey	Motorcycle riders—active crash
Özkan, Lajunen, Doğruyol, Yıldırım & Çoymak, 2012* †	-0.01	0.72	0.63	3	451	33.9	Turkey	Motorcycle riders –passive crash
Perry, 1986	0.46	1.3	-	-	54		US	University students
Poó, Taubman-Ben-Ari, Ledesma & Díaz-Lázaro, 2013*	0.25	0.79	0.39	2	642	39	Argentina	General drivers- Study 1
Poó, Taubman-Ben-Ari, Ledesma & Díaz-Lázaro, 2013*	0.04	0.3	0.17	1	258	35	Argentina	General drivers –Study 2
Reimer, D’Ambrosio, Coughlin, Kafrissen & Biederman, 2006	0.20	-	-	5	41	30.4	US	Drivers with ADHD and control group
Richer & Bergeron, 2012*	0.43	0.55	1.33	3	395	29	Canada	General drivers
Roach, Taylor & Dawson, 1999	0.36	0.5	-	1	133	19.6	Australia	Young drivers—Speed Camera
Roach, Taylor & Dawson, 1999 †	0.23	0.5	-	1	133	19.6	Australia	Young drivers –Police interception
Roskova, 2013*	0.24	0.44	1.46	3	531	33.4	Slovakia	General drivers
Schwebel, Severson, Ball & Rizzo, 2006*	0.07	0.37	1.03	2	73	27.8	US	University students
Schwebel, Ball, Severson, Barton, Rizzo & Viamonte, 2007*	0.45	2.1	2.2	5	101	80	US	Older drivers
Scialfa, Ference, Boone, Tay & Hudson, 2010*	0.07	0.14	0.3	2	73	73	Canada	Older drivers
Scott-Parker, Watson & King, 2009	0.20	0.2	0.33	3	165	19.7	Australia	Young drivers
Scott-Parker, Watson & King, 2010*	0.18	0.73	0.81	1.5	761	19	Australia	Young drivers
Scott-Parker, Watson, King & Hyde, 2013*	0.05	0.03	0.04	1	1,048	18	Australia	Young drivers
Šeibokaitė, Endriulaitienė, Žardeckaitė-Matulaitienė & Markšaitytė, 2011*	0.13	0.33	1.45	1	40	21.7	Lithuania	Young drivers
Smith & Heckert, 1998	-0.18	0.9	0.6	3.5	76	20	US	University students
Smith & Kirkham, 1982	0.19‡	-	-	3	113	-	Australia	Young male drivers
Sobel & Underhill, 1976	0.22	-	-	2	283	-	US	Male drivers
Sobel & Underhill, 1976	0.11	-	-	2	213	-	US	Female drivers
Stephens & Groeger 2009	0.07	0.29	0.19	3	47	25	UK	General drivers and university students
Stephens & Sullman, 2014 * †	0.21	0.18	1.15	1	551	37.9	UK	General drivers -–minor crashes
Stephens & Sullman, 2014 *	0.31	0.03	1.15	1	551	37.9	UK	General drivers -–major crashes
Stradling, Meadows & Beatty, 2004	0.07	0.22	-	3	791	-	UK	General drivers
Sullman & Stephens, 2013* †	0.50	0.09	0.06	0.25	213	44	NZ	General drivers—minor crashes
Sullman & Stephens, 2013*	-0.01	0.01	0.06	0.25	213	44	NZ	General drivers—major crashes
Sullman, Stephens & Kuzu, 2013*†	0.05	0.18	0.16	0.25	245	43.4	Turkey	Taxi drivers—minor crashes
Sullman, Stephens & Kuzu, 2013*	0.13	0.04	-	0.25	245	43.4	Turkey	Taxi drivers– major crashes
Sullman, Stephens & Yong, 2014*	0.14	0.32	0.23	0.25	339	26.6	Malaysia	General drivers
Sucha, Sramkova & Risser, 2014*	0.37	0.39	0.46	8	2,684	26	Czech	General drivers
Sümer, Ayvaşik, Er & Özkan, 2001	0.31	0.93	0.94	3	79	30	Turkey	General drivers and taxi drivers
Siimer, Lajunen, & Ozkan, 2005	0.30	0.88	1.49	3	1,001	36.3	Turkey	General drivers
Taylor & Sullman, 2009*	0.02	0.44	0.31	1	301	23.7	NZ	University students
Vingilis, Seeley, Wiesenthal, Mann, Vingilis-Jaremko, Vanlaar & Leal, 2013*	0.24	0.28	0.24	5	501	39	Canada	Racing car enthusiasts. Traffic offence mean is for 1 year period.
Whissell & Bigelow, 2003	0.31	0.24	-	2	255	20.8	Canada	Young drivers
Wishart, Freeman, Davey, Wilson & Rowland, 2012	0.15	0.16	0.1	2	546	44	Australia	Fleet vehicle drivers
Wishart, Freeman, Davey, Rowland, & Barraclough, 2014	0.12	0.12	0.1	1	3,414	42.8	Australia	Fleet vehicle drivers
Wishart, Freeman, Davey, Rowland, & Barraclough, 2014*†	0.09	0.1	0.18	1	3,397	42.8	Australia	Private vehicle usage
Wu, Aguero-Valverde & Jovanis, 2014*	-0.09	1.28	1.49	1	90	36	US	General drivers

* Unpublished data supplied by study author

^†^ Not selected for final meta-analysis

^‡^ Effect calculated from data in paper.

**Table 3:** Data from archival road safety studies showing moderating variables and sample characteristics.

Study	Correlation	Crash Mean	Traffic Offence Mean	Time Period (in years)	N	Age	Country	Sample
Barkley, Murphy, Du Paul & Bush, 2002* †	0.30	0.6	5.1	4.5	63	21.1	US	Young drivers with ADHD
Barkley, Murphy, Du Paul & Bush, 2002*	0.55	0.4	2.1	4.8	63	21.2	US	Young drivers
Burg, 1968	0.26	0.16	0.53	3	2,944	40	US	General drivers -Female
Burg, 1968	0.29	0.30	1.52	3	4,897	40	US	General drivers—Male
Burg,1971†	0.35	-	-	6	17,769	-	US	General drivers
Burns & Wilde, 1995 **	0.29	1.7	2	8	51	-	Canada	Taxi drivers -Traffic Violations excludes speeding
Burns & Wilde, 1995 **	-0.05	1.7	2.6	8	51	-	Canada	Taxi drivers—speeding fines
Chapman, Masten & Browning, 2014*	0.19	0.34	1.05	3	1,709,342	20.2	US	Novice drivers
Coppin & McBride, 1965	0.26	0.15	0.85	3	94,935	39.4	US	General drivers—California Driver Study, 1958 data
Coppin & McBride, 1965	0.27	0.20	0.54	3	148,000	38.3	US	General drivers -California Driver Study, 1965 data
Daigneault, Joly & Frigon, 2002	0.10	0.12	0.32	3	187,620	67	Canada	Older drivers Aged 65–69 –Predictive methodology
Daigneault, Joly & Frigon, 2002	0.10	0.13	0.24	3	131,334	72	Canada	Older drivers Aged 70–74 –Predictive methodology
Daigneault, Joly & Frigon, 2002	0.10	0.15	0.19	3	71,637	77	Canada	Older drivers Aged 75–79 –Predictive methodology
Daigneault, Joly & Frigon, 2002	0.08	0.18	0.15	3	3,818	82	Canada	Older drivers Aged 80+—Predictive methodology
Diamantopoulou et al 1997	.02‡	0.01	0.35	2	3,494,307	40	Australia	General drivers—Predictive methodology
Edwards, Hahn & Fleishman, 1977	0.28	-	-	5	152	-	US	Taxi Drivers
Factor, 2014	0.06	0.06	2.03	7	409,051	37.9	Israel	General drivers
Ferdun, Peck & Coppin, 1967	0.15	0.13	-	1	3,385	18	US	Male teenage drivers
Ferdun, Peck & Coppin, 1967	0.16	0.13	-	1	2,255	18	US	Female teenage drivers
Gebers & Peck, 1994	0.16	0.29	-	6	114,618	-	US	General drivers
Harrington, 1972	0.26	0.35	0.83	4	5,790	-	US	Young female drivers
Harrington, 1972	0.29	0.64	3.17	4	8,000	-	US	Young male drivers
Keall & Frith, 2004	0.01‡	0.01	0.01	2	39,318	84	NZ	Older drivers
McGuire, 1956b	0.44	0.86	-	-	134	-	US	Military personnel
Owsley, Ball, Sloane, Roenker & Bruni, 1991	0.34	0.32	-	5	53	-	US	Older drivers
Peck, McBride & Coppin, 1971	0.12	0.88	-	1	86,726	-	US	Male drivers -1961 data
Peck, McBride & Coppin, 1971	0.12	0.09	-	1	86,726	-	US	Male drivers -1962 data
Peck, McBride & Coppin, 1971	0.1	0.08	-	1	86,726	-	US	Male drivers—1963 data
Peck, McBride & Coppin, 1971	0.07	0.04	-	1	61,280	-	US	Female drivers -1961 data
Peck, McBride & Coppin, 1971	0.07	0.04	-	1	61,280	-	US	Female drivers—1962 data
Peck, McBride & Coppin, 1971	0.07	0.04	-	1	61,280	-	US	Female drivers—1963 data
Peck & Kuan, 1983	0.17	0.34	0.45	3	87,908	-	US	General drivers
Peck, 1993 †	0.35	-	-	14	78,742	-	US	General drivers—Major Violations
Peck, 1993†	0.20	-	-	14	78,742	-	US	General drivers—Speeding violations
Sakashita, Senserrick, Lo, Boufous, Rome & Ivers, 2014*	0.05	0.04	0.15	1.2	1,305	36	Australia	Motorcycle riders—Police reported crash
Shope, Waller, Raghunathan & Patil, 2001	0.28	0.44	1.06	7.15	2,070	23	US	Young male drivers
Shope, Waller, Raghunathan & Patil, 2001	0.21	0.26	0.41	7.15	2,332	23	US	Young female drivers
Smith, 1976 ‡	0.19	0.92	0.88	3	113	21.5	Australia	Young male drivers
Stewart, 1957	0.17‡	0.19	-	-	275	-	US	General Drivers—Speeding citations
Stewart & Campbell, 1972	0.13‡	0.13	0.23	2	2,500,448	40	US	General drivers—Predictive
Trimpop & Kirkcaldy, 1997	0.43	1.11	-	5.2	120	22.5	Canada	Young male drivers
Waller, Elliott, Shope, Raghunathan & Little, 2001	0.37	1	2.64	7.1	13,809	23.4	US	Young drivers
Wasielewski, 1984	0.29	-	-	7	2,561	45	US	General drivers
Wilson & Jonah, 1988	0.31	-	-	3	935	-	Canada	General and risky drivers

* Unpublished data supplied by study author

** Figures from paper combined for final meta-analysis

^†^ Not selected for final meta-analysis

^‡^ Effect calculated from data in paper.

An additional nine effects were identified from seven studies which compared variables created utilizing a combination of self-report and archival data and are shown in Table 4 [40,63,86,120,121,153]. All but two of these effect types were supplied by the study authors. These effects were not included in the meta-analysis.

**Table 4:** Combining or cross matching self-reported and archival crash and traffic offence variables with moderating variables and sample characteristics.

Study	Effect	Crash Mean	Traffic offence mean	Time period (in years)	N	Age	Country	Sample
Barkley, Murphy, Du Paul & Bush, 2002*	0.18	1.9 (self-report)	0.73 (archival)	4.5	105	21	US	Young drivers with ADHD
Barkley, Murphy, Du Paul & Bush, 2002*	0.28	1.2 (self-report)	0.34 (archival)	4.8	63	21	US	Young drivers control group
Caird, 2004	0.23	0.56 (archival)	0.29 (self-report)	2	153	42	Canada	Working drivers
Fischer, Barkley, Smallish & Fletcher, 2007*	0.11	1.88 (self-report)	6.12 (archival)	3.6	86	21.1	US	Young drivers with ADHD
Fischer, Barkley, Smallish & Fletcher, 2007*	0.16	1.61 (self-report)	3.22 (archival)	4.2	46	20.5	US	Young drivers—control
Sakashita, Senserrick, Lo, Boufous, Rome & Ivers, 2014*	0.01	0.25 (self-report)	0.15 (archival)	1.2	651	36	Australia	Motorcycle riders
Smith, 1976 ‡	0.13	0.92 (combination)	0.88 (combination)	3	113	21.5	Australia	Young male drivers
Schwebel, Ball, Severson, Barton, Rizzo & Viamonte, 2007*	0.45	0.15 (archival at fault)	2.2 (self-report)	5	101	80	US	Older drivers–“at fault crashes”
Schwebel, Ball, Severson, Barton, Rizzo & Viamonte, 2007*	0.43	0.33 (archival)	2.2 (self-report)	5	101	80	US	Older drivers

* Unpublished data supplied by study author

^‡^ Effect calculated from data in paper.

### Outlier detection

Outlier detection was conducted separately for self-report data and the archival studies. Figures were converted to reflect an annual means. Table 5 shows Uni-variate and Bi-variate data identified as outliers. In relation to self-reported crash means, the use of a relatively broad definition of crashes in some of the studies may partially explain these figures. The exclusion of these outliers did not affect the results of subsequent analyses (results not shown) and, consistent with Glass [57], who advocates the inclusion of as many relevant studies as possible, the reported analyses include all studies indicated above.

**Table 5:** Uni-variate and Bi-variate outliers.

Crash means		Traffic offence means		Bi-variate outliers	
Self-report	Archival	Self-report	Archival offence	Self-report	Archival
Ouimet et al., (2008) Sullman, Stephens & Kuzu, (2013) Sullman, Stephens & Yong (2014) Wu, Aguero-Valverde & Jovanis (2014)	Peck, McBride & Coppin, 1971)—1961 sample of male drivers only	Barkley et al., (2002)—ADHD sample Conner & Lai (2005)—both samples	Barkley et al., (2002)—ADHD sample Burns & Wilde (1995) Harrison (1972)—male drivers	Galovski & Blanchard (2002) McGuire (1956) Özkan & Lajunen (2005) Schwebel et al., (2007)	Burns and Wilde (1995)

### Descriptive data

An overview of the means of key variables is shown in Table 6. When interpreting the figures in this section, it is important to bear in mind that the results are based on analysis of the means of study variables, as opposed to representing an individual’s relationship between variables, as is contained within each actual study. For example the strong positive correlation found between crash means and the mean of traffic offences (see Table 7) simply shows that studies that report a higher crash mean tend to also report higher means for traffic offences. In addition, the results in this section are not weighted for sample size unlike in the Meta-regression that follows. All correlations are statistically significant unless otherwise indicated.

**Table 6:** Means of variables of interest.

Variable	Self-report	Archival
Correlation between crashes and offences	.18 (SD .16)	.20 (SD.13)
Crash mean	.59 (SD .60)	.38 (SD .43)
Annualised crash mean	.24 (SD .23)	.11 (SD .15)
Traffic offence mean	1.16 (SD 1.60)	1.18 (SD 1.22)
Annualised traffic offence mean	.50 (SD .68)	.28 (SD.25)
Mean sample size	821.51 (SD 1415)	216, 522 (SD 667677)
Mean age of study participant (in years)	33.5 (SD 12.4)	39.6 (SD 21.3)
Time period under investigation (in years)	2.91 (SD 2.8)	3.93 (SD 2.72)

**Table 7:** Pearson correlations between the key variables (without controlling for time period under investigation). Number of studies used in the analysis (k) is shown in parenthesis.

	1	2	3	4	5	6	7
1. Effect (correlation)	—	.43** (129)	.47** (104)	-.10 (144)	-.24* (109)	.27** (134)	-.20* (144)
2. Crash mean		—	.63** (104)	-.14 (129)	-.22 (106)	.50** (122)	-.12 (129)
3. Offence mean			—	-.08 (104)	-.27** (96)	.23* (103)	-.11 (104)
4. Sample Size				—	.03 (109)	-.04 (134)	-.07 (144)
5. Age					—	-.09 (108)	.10 (109)
6. Time period under investigation (in years)						—	-.01 (134)
7. Year of publication							—

*p < .*05*,

** *p <* .*01*.

In this instance the item “Correlation between crashes and offences” represents the mean measure of association obtained for the relationship between crashes and traffic offences within the examined studies and not a figure calculated subsequently accounting for moderators. The item “time period” refers to the length of time over which crash and offence history was collected in each study. As shown in Table 6, self-report studies tended to contain higher crash means despite the shorter average time frame over which study participants’ traffic history was examined. This finding reflects the degree to which the measurement of crashes, as recorded by archival records is likely to be limited to more severe crashes, i.e. subject to under-reporting of minor crashes. Annualised averages for the crash variable were .24 (SD .63, k = 73) for self-reported data and 0.11 (SD .15, k = 35) for archival records.

An analysis was undertaken to assess whether the mean number of crashes may be associated with correlations between crashes and offences. After controlling for time period under investigation, a moderate positive correlation between crash mean and mean measure of association size was detected (*r* = .33, k = 120). This relationship was more pronounced in regards to self-reported studies (*r* = .40, k = 84) than those using archival records (*r* = .08, ns, k = 34). A similar analysis to examine the relationship between the traffic offence mean and the correlation between crashes and offences produced similar results. After controlling for time period under investigation, the correlation between the mean number of recorded offences and the mean measure of association size was r = .42 (k = 101). Again this result was more apparent in self-reported studies (r = .43, k = 76) than in the archival data (r = .36, ns, k = 23).

Of the three self-report studies which reported separate correlations for minor and major crashes two observed stronger correlations for major crashes (Stephens & Sullman, 2014; Sullman, Stephens & Kuzu, 2013). The difference in means for both crashes and time period between self-reports and archive data underscores the necessity of controlling for these items if examining differences between these two data types. Similarly the slightly lower correlation for self-reported effect sizes must be interpreted in light of the shorter time periods under investigation.

In Table 6, it can be observed that the mean for archival offences was similar to that of self-reports. However, as the time periods used differ, these values are not directly comparable. Annualised averages for the traffic offences variable were calculated, these being 0.50 (SD .68, k = 74) for self-reported offences and 0.28 (SD .25, k = 26) for archival records. An additional within country analysis was undertaken in which only the means from countries which could provide both data types were considered. This showed that the discrepancy between the two offence measures was still present, with an annual rate of 0.46 traffic offences (SD .53, k = 36) for self-reported data while the figure for archival data remained 0.28 (SD .27, k = 24).

### Bivariate relationships

A series of bivariate relationships were examined. Raw correlations between variables are presented in Table 7 with Table 8 providing a differentiation between self-reported and archival data. Controlling for time period, where appropriate, is a key component in the subsequent analyses. To highlight the importance of this aspect, occasionally results of an analysis are provided without controlling for this element.

**Table 8:** Pearson correlations between key variables showing both data types (without controlling for time period under investigation). Self-reported effects are shown in the top portion of the table while archival effects are shown below. Number of studies used in the analysis (k) is shown in parenthesis.

	1	2	3	4	5	6	7
1. Effect (correlation)	—	.47** (91)	.48** (79)	-.01 (99)	-.16 (85)	.19 (93)	-.22* (99)
2. Crash mean	.33* (38)	—	.65** (79)	-.23* (91)	-.12 (83)	.50** (86)	-.52** (91)
3. Offence mean	.43* (25)	.55** (25)	—	-.19 (79)	-.21 (76)	.18 (78)	-.35** (79)
4. Sample Size	-.26 (45)	-.21 (38)	-.22 (25)	—	.07 (85)	-.05 (93)	.16 (99)
5. Age	-.55** (24)	-.47* (23)	-.53* (20)	-.04 (24)	—	-.09 (84)	.39** (85)
6. Time period under investigation (in years)	.45** (41)	.62** (36)	.51** (25)	-.17 (41)	-.24 (24)	—	-.04 (93)
7. Year of publication	-.11 (45)	.09 (38)	.06 (25)	.14 (45)	.21 (24)	.38* (41)	—

*p < .05,

** *p* < .01.

#### Effect size and time period

Overall the correlation between mean measure of association size and time period was .27, indicating that the longer the time period under investigation, the greater the strength of the correlation between crashes and traffic offences. However for self-reported data this relationship was noticeably lower (*r* = .19, *p* = .07, k = 93) than that recorded by archival studies (*r* = .45, *p* = .003, k = 41). It is possible that the younger mean age of study participants in self-report studies may explain this discrepancy, i.e. younger drivers have more crashes and this may produce comparatively strong correlations in shorter time periods. However a series of partial correlations did not support this hypothesis. After controlling for participant age, the overall and archival correlations remained significant, (*r* = .22, k = 106) and (*r* = .43, k = 22) respectively, while the relationship as recorded by self-report data was also relatively unchanged (*r* = .18, ns, k = 82).

#### Effect size and age

The association between participant age (mean age in sample) and measure of association was negative indicating that stronger correlations between crashes and offences were generally found in younger drivers. After controlling for time period, the overall correlation remained unchanged and statistically significant, (*r* = -.24, k = 106). However the relationship as recorded by self-report data was weaker (*r* = -.17, ns, k = 82) while the archival studies, revealed a much stronger outcome (*r* = -.51, *p* = .01, k = 22). Comparable results were obtained after controlling for crash mean and for offence mean, both on their own or in conjunction with the time period (results not shown).

#### Effect size and year of publication

This analysis considers whether the magnitude of measured effects is changing over time. Usually, such a trend would be considered to be indicative of dissemination bias, but in the present context it might also provide some insight as to possible changes in driving behaviour, as measured by the variables in this study. It should be noted that items making up the “year of publication” variable include previously unpublished data that was provided by researchers upon request by the study authors. Comparing the year of publication with effect sizes produced a weak negative relationship (*r* = -.20, *p* = .02, k = 144). Controlling for crash and offence means and time period under investigation produced a similar result (*r* = -.20, *p* = .05, k = 99). This finding suggests that reported measures of association between crashes and traffic offences are becoming somewhat weaker over time.

Another examination of the relationship between effect size and year of publication was conducted with reference to the different data sources. After controlling for time period only, both data types yielded a slightly stronger effects; self-report (r = -.22, p = .04, k = 91); and archival (r = -.27, ns, k = 39). An additional analysis of the archival data, controlling for crash and offence means and time period produced stronger results (*r* = -.42, *p* = .05, k = 21). There is no evidence to suggest that publication bias is a factor in these findings, given that the relationship between crashes and offences was generally not a major focus of the studies from which data was retrieved. An alternate explanation could be a possible decline in crashes over time. Some support for this was found within the data. After controlling for time period and participant age, there was a noted decline in crash means reported in self-report studies (*r* = -.27, *p* = .015, k = 80) although this was not observed in the archival studies (*r* = -.03, ns, k = 20). A similar analysis of the self-reported means of traffic offences found that, after controlling for time period and participant age, these were seen to decline over time (*r* = -.26, p = .02, k = 73) more so than archival records (*r* = .13, ns, k = 17). However after controlling for crash mean only, slightly stronger measures of association were observed in the archive data (*r* = -.15, ns, k = 36) than that of self-report (*r* = -.03, ns, k = 89).

So reductions in effect size, crash mean and offence mean in regards to self-reported studies were observed over time. Conversely the associations between traffic offences and crashes, as reported by archival studies, appear to be declining despite the absence of any discernible change in the frequency of reported crash or traffic offences.

#### Effect size and Sample size

No significant effects were obtained in relation to sample size and the correlation between crashes and traffic offences. After controlling for time period, both self-report and archival data produced weak results (*r* = -.08, ns, k = 91) and (*r* = -.20, ns, k = 39) respectively.

#### Crash mean and offence mean

As noted earlier, the relationship between crash means and offence means (*r* = .63 *p* < .001, k = 104) describes the association between recorded means within studies and does not reflect the actual correlations between individual crashes and offences that is the focus of the following section. After controlling for time period, the overall result was still strong (*r* = .53 < .001, k = 101), though more pronounced for self-report studies (*r* = .57 < .001, k = 76) than archival data (*r* = .32, ns, k = 23).

### Mean effects and Meta-regression

#### Data

A total of 128 studies were selected as being suitable for inclusion in these analyses in line with the selection criteria outlined earlier. Of these, 86 studies provided measures of association from self-reported data. This self-report component represents a total sample size of 68,097 drivers drawn from studies in 20 different countries. Forty-two archival studies were included, representative of a total of 9,489,647 drivers. Archival data was obtained from five countries: The United States; Canada; Australia; New Zealand and Israel.

#### Mean effects

An average measure of association between crashes and offences, after weighting for sample size, was calculated, *r* = .18, 95%CI [.17, .20] *p* < .001. The average time period for these studies was 3.23 years.

To facilitate a meaningful comparison of findings drawn from self-report and archival data, particularly to examine for possible CMV effects, studies from regions for which both data types were available were examined utilising a “within country” approach, thus excluding between countries variance. This involved three analyses, focusing separately on data from the US, Canada and a combination of studies from Australia and New Zealand. For the purposes of this analysis data from Australia and New Zealand was treated as being sourced from a single jurisdiction, given the similarities between the two countries in regards to road traffic laws, driving environment and enforcement practices [154].

As shown in Table 9, the confidence intervals for the different data types from Canada, Australia and New Zealand do not overlap. In the case of data from the US, the lower limit of the confidence interval for self-report mean measures of association and the upper limit of archival mean measures of association are both .23. To ascertain the degree to which the findings from the other countries may be present in the US data a series of sensitivity tests, excluding studies for which the period under examination was six or more years was conducted, in part because it was not possible to satisfactorily control for the time period in this particular analysis. This analysis produced results consistent with the other countries for which both data types were available, i.e. stronger effects observed in relation to self-reported data.

**Table 9:** Within country comparison of effects from self-report and archival studies utilising a random effects analysis.

Country and data type	Number of effects	Point estimate	Total sample size	95% Confidence intervals		Z-value	P-value
				Lower limit	Upper limit		
Canada Self-report	4	.29	1,275	.16	.41	4.28	< .001
Canada Archival	7	.11	395,464	.10	.13	13.70	< .001
Australia New Zealand Self-report	15	.14	13,776	.11	.16	12.07	< .001
Australia New Zealand Archival	4	.03	3,535,043	.00	.05	1.96	.050
US Self-report	31	.26	11,997	.23	.29	14.85	< .001
US Archival	29	.21	5,307,471	.19	.23	17.99	< .001
US Sensitivity analysis Self-report *	26	.26	11,432	.23	.30	14.37	< .001
US Sensitivity analysis Archival*	26	.20	5,172,081	.17	.22	15.35	< .001

* Excluding studies longer than 6 years.

As there were differences in crash means between countries as well as between sources, and to make comparisons with other crash predictors possible, a calculation was made to determine what the effect would be in a sample with a crash mean of 1. This method used the regression formula for the correlation between effect sizes and crash means in the samples. However, as some of the sub-samples in Table 9 were very small, and the regression formulas therefore very unstable, the data for all the countries in that table was pooled, and the correlations between crash means and self-report or archive data mean measures of association were computed. It was found that self-reports had an expected *r* of .26, and archives an expected *r* of .25 at crash mean 1.

#### Moderator analysis

To test for whether the source of the data influenced the strength of the measures of association after controlling for other moderators, meta-regressions were run with source of data, age, time period, crash and offence means as moderators. As there were some missing data in most variables, moderators other than source which were not found to explain a significant amount of variance were deleted from the model. Therefore, the results, shown in Table 10, only contain significant predictors, apart from source. If source is also a significant predictor, this indicates that the results in Table 9 are sound, i.e. that the difference between self-report and archival data in relation to the size of the effects persisted even after controlling for other known moderators.

**Table 10:** Results of meta-regressions with moderators (random models). Shown are the number of effects included (k), the moderators which added significantly to the model (p < .1), apart from the source of data, and the statistics for the model.

Country	Number of effects	Moderators	Coefficient	95% Confidence intervals		Z-value	p-value
				Lower limit	Upper limit		
Canada	7	Source	-0.228	-0.307	-0.148	-5.62	< .001
		Offence mean	0.081	0.010	0.152	2.25	0.02
		Time period	-0.044	-0.085	-0.003	-2.12	0.03
Australia & New Zealand	17	Source	-0.098	-0.138	-0.058	-4.81	< .001
		Offence mean	0.084	0.022	0.147	2.65	0.01
US	35	Source	-0.033	-0.089	.022	-1.18	0.24
		Offence mean	0.049	0.032	0.066	5.80	< .001

It can be seen that the results are in good agreement, with strong differences between sources for Canada and Australia/New Zealand, and a small and uncertain one for the US. Differences in the known moderator variables do therefore not explain the difference between sources.

## Discussion

The current paper has endeavored to explore the relationship between crashes and traffic offences giving consideration to possible influential elements including age, sample size, time period under investigation, regional factors and the type of data used. The nature of the correlations observed suggests that generally the relationship between crashes and traffic offences is not strong. The relationship presumed to be present between these variables may in fact be rather tenuous, or in many instances the associations detected may actually reflect other elements, such as exposure to the road. The weakness of the relationship between these variables suggests that the effectiveness of using traffic offences as a proxy for crashes in road safety studies is very limited (as argued by af Wåhlberg [1]) with implications for researchers and policy makers. This conclusion is in agreement with the finding that driver education programs often have a mixed influence on crashes and offence rates, usually by reducing only the latter [155]. Also, the degree to which crashes are attributable to the unsafe behaviour of the individual is generally not measured in many road safety surveys. In such instances, the number of traffic offences, or general assessments of driving behaviour may be irrelevant if a subsequent crash is the fault of a different driver altogether. There is also no reason to expect the offences-crashes correlation to be high, as both variables contain a large degree of randomness. For a crash to occur, or an offence to be registered, several different circumstances must interact. Accordingly, neither variable may be strongly connected to actual behaviour.

The correlation between offences and crashes was found to decline somewhat over time (i.e. with year of publication), a phenomenon which is not unknown in the scientific literature [46,156–158]. Three possible explanations would seem to exist for this. First, it is possible that this is caused by a trend in dissemination of results, i.e. a publication artefact. Second, it might be due to changes in driving behaviour over time as it relates to the measurement of crashes and traffic offences. Third, changes in legislation and enforcement may alter the types and frequencies of offences detected. For example the increased use of automated enforcement technology, such as speed cameras, would capture more offences of this type. Prior to this, it is possible that the detection of more blatant devious driving behaviour made up a larger proportion of traffic offences and that these offences were more indicative of potential crash involvement. Which of these explanations, if any, is correct is not possible to ascertain from the data available here.

In the total dataset, mean age in the samples was negatively related to the strength of the measure of association between crashes and offences, an association which was not explained by other moderators, such as the crash mean. However, the relationship was found exclusively in the archival data. This difference could reflect the degree to which archival studies tend to capture more severe crashes. This crash type is understood to become less frequent with age relative to minor crashes. Certainly younger drivers tend to have more severe crashes than older drivers [159,160]. Accordingly, the association between severe crashes and traffic offences could diminish as they become relatively less common.

Evidence for CMV effects (i.e. larger effects for self-reported data) was present to some degree in our data. These results point to a pronounced difference in terms of findings from self-report effects and archival data. In all countries for which both data types were available, self-reported data produced stronger mean measures of association. This finding is consistent with the concern that inflated effect sizes may occur when all data is from a single source, particularly if it is self-reported data. We therefore predict that when archival data becomes available for those countries for which at present only self-reports are available, the former will generally be smaller. However it must also be noted that the differences between data types, though statistically significant, was not particularly large, especially in the calculation of the expected measures of association. The extent to which this difference accurately reflects the nature of the two data types or represents a distortion due to CMV effects remains uncertain. However the current study highlights the risk that inflation of effect sizes, even if minor, can distort results. Findings in which effect sizes are small but of interest to researchers due to the relative rareness of the dependent variable, may be sensitive to CMV effects. Accordingly this should be a consideration in studies involving the prediction of vehicle crashes [46]. While the results described in the current study suggest that self-reported data from a single source can lead to an overestimation of the effect size, it is important to note that biases potentially present within archival records and the lower number of crashes recorded, could lead to under-estimations of the effect size. Nevertheless, a difference was detected in effect size between sources even after controlling for accident mean.

The CMV analysis by country also indicates that these effect sizes are to some extent country specific and probably reflect differences in law and surveillance practices. This suggests that analysis of the offence/crash association is best conducted examining each country separately. Some of the outlying effects identified in this study are also probably explained by differences between cultures and countries, as several of them were from countries with few studies published, and would thus be different from the majority of data. Similarly, some outliers were reported from special populations. For example, in Barkley, Murphy, DuPaul and Bush [63] the mean of offences was very deviating in both univariate and bivariate analyses. What makes this result especially interesting was the fact that offences were available both from records and self-reports, and both sources yielded these outliers. This indicates that this was not some sort of error in the paper, but due to something else, probably the special population studied, in this instance young drivers with ADHD.

Longer time periods of investigation did not produce stronger measures of association in self-report data, indicating that memory effects or some other bias are in play. Consistent with this, the correlation between accident mean and time period was stronger for archive data. Other factors, such as exposure to the road, the actual traffic laws in place and the degree to which they are enforced undoubtedly contribute to the relationship between crashes and traffic offences.

The difference in crash means between the two data types is not unexpected, given that self-reported crashes allow for a wider range of crash types to be recorded. However a comparison of the mean number of traffic offences is noteworthy given that this measure should basically be the same for the two data types. The overall annualised means, 0.50 for self-reported and 0.28 for archival records, representing a considerable difference, indicates that participants tend to self-report many more traffic offences than recorded in archival data. This curious discrepancy has been noted in previous research [161]. In the current study one possible explanation is that archives have been purged of older citations. However, this would mean that the time period used would correlate negatively with the mean number of offences per year for records, but not for self-reports. However, the opposite was found in the present data, indicating a memory loss effect in self-reports. As much of the data was from countries with federal states, it is also possible that drivers self-reported incidents that occurred in different jurisdictions, i.e. interstate, than those for which archival data has been sourced. However, limiting the analysis to such countries did not produce a larger difference. Another factor could be the extent to which infringement notices are issued to individuals who did not incur the penalty. For example a fine from a speed camera may be issued to the registered owner of a vehicle rather than the actual driver. However it would be expected that such instances would tend to balance themselves out in the data and in any event would not explain the discrepancy observed in this analysis. It is also possible that in some instances drivers mistakenly report offences that they committed but were not detected by authorities. However it is generally understood in the road safety domain that questions in surveys relating to traffic offences in a driving history refer to actual cases of illegal behaviours for which they had been caught and a subsequent fine or penalty issued. This phenomenon is therefore still unexplained, although it is suggested that this is some sort of recall effect, which is in need of further research.

Differences between self-reported and archival data were also found in several other instances, which were not predicted, and which are difficult to interpret. For example, the decline in effect size in studies with increasing age of the sample was only present in recorded data. Such effects, although currently somewhat mysterious, also point to the basic difference between self-reported and recorded data. These sources are prone to yielding different results, and we often do not know exactly why.

### Limitations

While every effort was made to collect and control for items that may influence the relationships observed in this study, it must be acknowledged that exposure to the road also plays a role in the frequency of road incidents and this would in turn influence the observed variance. Some findings from the current study, such as the observed relationship between crash means and offence means no doubt also reflect this issue. Relatively few studies include a measure of the amount of time participants spend behind the wheel. Indeed this aspect would be impossible to capture through state sources although some fleet vehicle records would have relevant exposure data. Some previous road safety findings have been found to be strongly influenced by exposure with those who drive more often also having a greater risk of experiencing an adverse driving event, although this relationship is not always so straight forward [47,152,162]. Conversely, repeated traffic infringements could lead to licence disqualification which in turn would reduce exposure to the road, directly reducing the likelihood of crash involvement. In this case the predictive capacity of traffic offences would be diminished or even negated. Also, longer periods of licence disqualification, associated with more mature drivers, may in part explain the observed decline in effect sizes with age although it must also be noted that disqualified drivers who continue to drive have a higher crash risk than licensed drivers [163,164]. Ideas associated with deterrence theory may also be reflected in the data. While, it is certainly possible that the experience of a crash might also promote safer driving and consequently fewer offences and vice-versa, the evidence for such behaviour is mixed with crash records found to be relatively stable over time [1,165,166]. The impact of the exposure factor on the association between offences and crashes in terms of underlying behaviour may be considerable. If the correlation between these variables, as calculated here, is to some degree explained by exposure, it can indeed be questioned whether these variables do have a commonality of any significant or practical degree. As noted previously, recordings of crash involvement tend not to indicate whether the driver was at fault. In such instances, the number of traffic offences, or general assessments of driving behaviour may be irrelevant if a subsequent crash is the fault of a different driver altogether. Road safety variables, particularly traffic offences, could simply reflect exposure to the road.

Another issue to arise was the treatment of results from predictive and postdictive methodologies. Predictive study designs utilise data that are obtained prior to an event of interest and then assesses these measures against criteria obtained at a later date. Postdictive designs are much more common, particularly in road safety. This study type involves the analysis of data relating to both independent and dependent variables measured during the same time period. In the current study only three predictive studies were identified, all using archival data. Predictive designs may produce results that differ from those obtained by the more commonly-used postdictive methods [167] however as noted earlier, none of these studies appeared as outliers in preliminary analysis.

### Future research

Future research would be enhanced by the inclusion of more studies using archival records, particularly from within Europe. This would provide a more meaningful point of reference to the relatively large range of data from self-reported sources. This would also facilitate a more thorough assessment of the differences the two data types can produce. In addition road safety researchers may consider including a measure of both major and minor crashes to facilitate comparisons of accident type. These variables could easily be combined later to produce the variable commonly used in other components of the analysis. The correlations produced by the different types of crash and offence variables were not identified as being problematic by the outlier detection. However as more studies become available the use of a more restricted definition of these variables may be possible in any future recreation of this analysis. Also, differences between countries and special populations could be suspected to yield differences in effects. In essence, future research should not treat the crash/offence association as surfacing from a unitary population. Ongoing research would also benefit from the inclusion in road safety studies of pertinent data as a matter of course, particularly exposure data. Information relating to items examined within a study, such as means, test statistics and correlation matrixes, would allow researchers, particularly those conducting a meta-analysis, to better assess the impact of potential moderator variables. Future research would be also be enhanced if the degree to which reported crashes are due to the unsafe behaviour of the individual could also be investigated. In addition it would be of interest to further explore the extent to which traffic infringements act as a deterrent to dangerous behaviour. The use of data sets containing very comprehensive driver histories could examine the degree to which traffic offences occur following a crash or conversely whether or not crash involvement leads to fewer subsequent driving offences. Finally, conducting more meta-analyses that focus on aspects of crash prediction, for example fatigue, would further illuminate the impact of various factors on road safety.

### Overall conclusion

The aims of the study were realised. After weighting for sample size, an average correlation between crashes and offences of *r* = .18 was observed over a mean time period of 3.2 years. Consistent with common method variance effects, a within country analysis found stronger effect sizes in self-reported data even after controlling for crash mean, however these differences were small.

## Supporting Information

S1 PRISMA Checklist 2009 Checklist. (PDF)
